# 310. A comparative study on the effectiveness and safety between colistin-based and high-dose ampicillin/sulbactam-based combination therapy for nosocomial pneumonia caused by carbapenem-resistant *Acinetobacter baumannii*

**DOI:** 10.1093/ofid/ofae631.100

**Published:** 2025-01-29

**Authors:** Jaehoon T Lee, Imchang Lee, Ki-Byung Lee, Seung Soon Lee

**Affiliations:** Hallym University Chuncheon Sacred Heart Hospital, Chuncheon-Si, Kangwon-do, Republic of Korea; Hallym University Chuncheon Sacred Heart Hospital, Chuncheon-Si, Kangwon-do, Republic of Korea; Hallym University Chuncheon Sacred Heart Hospital, Chuncheon-Si, Kangwon-do, Republic of Korea; Hallym University Chuncheon Sacred Heart Hospital, Chuncheon-Si, Kangwon-do, Republic of Korea

## Abstract

**Background:**

There are differences between IDSA guidance and ESCMID guideline in the treatment of nosocomial pneumonia caused by carbapenem-resistant *Acinetobacter baumannii* (CRAB). We compared differences in treatment outcomes between the colistin-based and high-dose ampicillin/sulbactam-based regimen in CRAB pneumonia.

**Methods:**

A retrospective cohort study was conducted on adult patients diagnosed with nosocomial pneumonia (clinically defined pneumonia or pneumonia with specific laboratory findings according to the CDC/NHSN surveillance definition) by CRAB (with a meropenem MIC ≥16ug/ml, colistin MIC 1 ≤ug/ml, and ampicillin/sulbactam MIC ≥4ug/ml) in a university-affiliated hospital in Korea. The study enrolled those who received colistin-based regimen using a loading dose of colistin followed by a maintenance dose (June 2021 to May 2022) and high-dose ampicillin/sulbactam-based regimen using sulbactam 9g/day (October 2022 to September 2023). The primary outcome (all-cause mortality within 28 days) and secondary outcomes (including 14-day/28-day clinical success rates and 14-day/28-day kidney injury -RIFLE score) between the two groups were compared using a Log-binomial regression.

**Results:**

A total of 170 CRAB pneumonia patients were enrolled in the study, including 87 patients receiving colistin-based regimen and 83 patients receiving high-dose ampicillin/sulbactam-based regimen. The 28-day mortality rate in the ampicillin/sulbactam group (27.7%) was significantly lower than the colistin group (57.5%) (adjusted RR = 0.48, 95% CI 0.33-0.71). The 14-day and 28-day clinical success rates of the ampicillin/sulbactam group (50.6% and 68.7%) were both significantly higher than those of the colistin group (23.0% and 32.2%) (aRR = 2.20, 95% CI 1.42-3.42 and aRR = 2.13, 95% CI 1.52-2.99). Also, both 14-day and 28-day kidney injury in the ampicillin/sulbactam group (0.6 ± 1.2 and 0.7 ± 1.4) were significantly lower than those in the colistin group (0.9 ± 1.2 and 1.0 ± 1.3) (aRR = 0.69, 95% CI 0.52-0.91 and aRR = 0.68, 95% CI 0.47-0.99).

**Conclusion:**

In patients with CRAB nosocomial pneumonia, High-dose ampicillin/sulbactam-based combination therapy is a better treatment option because of its lower mortality, higher clinical success rate, and lower risk of kidney injury.

**Disclosures:**

**All Authors**: No reported disclosures

